# Sphingosine 1-phosphate receptor 2 promotes the onset and progression of non-alcoholic fatty liver disease-related hepatocellular carcinoma through the PI3K/AKT/mTOR pathway

**DOI:** 10.1007/s12672-023-00611-8

**Published:** 2023-01-11

**Authors:** Ganggang Wang, Xin Zhang, Zhijie Zhou, Chao Song, Wenzhi Jin, Hao Zhang, Weixin Wu, Yong Yi, Hengguan Cui, Ping Zhang, Xinyu Liu, Weiqiang Xu, Xiaowei Shen, Weixing Shen, Xiaoliang Wang

**Affiliations:** 1 Department of General Surgery, Qingpu Branch of Zhongshan Hospital, Fudan University, Shanghai, China; 2 Department of Hepatobiliary Surgery, Pudong Hospital, Fudan University, Shanghai, China; 3 Department of Liver Surgery and Transplantation, Liver Cancer Institute, Zhongshan Hospital, and Key Laboratory of Carcinogenesis and Cancer Invasion (Ministry of Education), Fudan University, Shanghai, China

## Abstract

**Purpose:**

Recent studies have revealed an increase in the incidence rate of non-alcoholic fatty liver disease-related hepatocellular carcinoma (NAFLD-HCC). Furthermore, the association of Sphingosine 1-phosphate receptor 2 (S1PR2) with various types of tumours is identified, and the metabolism of conjugated bile acids (CBAs) performs an essential function in the onset and development of HCC. However, the association of CBA and S1PR2 with NAFLD-HCC is unclear.

**Methods:**

The relationship between the expression of S1PR2 and the prognosis of patients suffering from NAFLD-HCC was investigated by bioinformatics techniques. Subsequently, the relationship between S1PR2 and the biological behaviours of HCC cell lines Huh 7 and HepG2 was explored by conducting molecular biology assays. Additionally, several in vivo animal experiments were carried out for the elucidation of the biological impacts of S1PR2 inhibitors on HCC cells. Finally, We used Glycodeoxycholic acid (GCDA) of CBA to explore the biological effects of CBA on HCC cell and its potential mechanism.

**Results:**

High S1PR2 expression was linked to poor prognosis of the NAFLD-HCC patients. According to cellular assay results, S1PR2 expression could affect the proliferation, invasion, migration, and apoptosis of Huh 7 and HepG2 cells, and was closely associated with the G1/G2 phase of the cell cycle. The experiments conducted in the In vivo conditions revealed that the overexpression of S1PR2 accelerated the growth of subcutaneous tumours. In addition, JTE-013, an antagonist of S1PR2, effectively inhibited the migration and proliferation of HCC cells. Furthermore, the bioinformatics analysis highlighted a correlation between S1PR2 and the PI3K/AKT/mTOR pathway.

GCDA administration further enhanced the expression levels of p-AKT, p-mTOR, VEGF, SGK1, and PKCα. Moreover, both the presence and absence of GCDA did not reveal any significant change in the levels of S1PR2, p-AKT, p-mTOR, VEGF, SGK1, and PKCα proteins under S1PR2 knockdown, indicating that CBA may regulates the PI3K/AKT/mTOR pathway by mediating S1PR2 expression.

**Conclusion:**

S1PR2 is a potential prognostic biomarker in NAFLD-HCC. In addition, We used GCDA in CBAs to treat HCC cell and found that the expression of S1PR2 was significantly increased, and the expression of PI3K/AKT/mTOR signalling pathway-related signal molecules was also significantly enhanced, indicating that GCDA may activate PI3K/AKT/mTOR signalling pathway by up-regulating the expression of S1PR2, and finally affect the activity of hepatocellular carcinoma cells. S1PR2 can be a candidate therapeutic target for NAFLD-HCC. Collectively, the findings of this research offer novel perspectives on the prevention and treatment of NAFLD-HCC.

## Introduction

Hepatocellular carcinoma (HCC) is classified as one of the most prevalent types of tumours across the globe, with high incidence and mortality rates. According to the latest statistical report [1], liver cancer is identified as the fourth most prevalent type of cancer and ranked as the second leading cause of tumour-related mortalities in China. According to a rough estimate, around 1 million individuals suffering from HCC will die in 2030. Additionally, HCC-related 5-year survival rate is quite low i.e., only 18%, second just to pancreatic cancer in terms of fatality [2]. The primary causes of liver cancer are viral hepatitis, heavy alcohol consumption, and non-alcoholic fatty liver disease (NAFLD) [3]. The promotion of vaccination and the improvement of hygienic practices in recent years have remarkably reduced the incidence of viral hepatitis [4].Between 1990 and 2019, the overall global prevalence of NAFLD was about 30 per cent. However, during this period, the prevalence of NAFLD increased at an annual rate of 0.7%, reaching 37% by 2019 [5].Due to different etiology and biological mechanism, the clinical characteristics and prognosis of patients are different. The mechanisms underlying the onset and progression of NAFLD-HCC are still unclear. Therefore, relevant studies are needed to elucidate the prevention and treatment of NAFLD-HCC.

Sphingosine 1-phosphate (S1P) is a biologically active lysophospholipid, with different physiological effects on various types of cells and tissues. S1P receptors are a group of G protein-coupled receptors, classified into five isotypes: S1PR1, S1PR2, S1PR3, S1PR4, and S1PR5. S1P receptors are expressed in a variety of tissues and exhibit different cellular specificities. The human S1PR2 gene is located on chromosome 19 (19p13.2), and the protein encoded by this gene acts as a cell membrane receptor for S1P [6]. Moreover, S1PR2 functions in tumours through different pathways, including the JAK/STAT pathway [7], NF-KB pathway [8], ERK signalling pathway [9], and PI3K/AKT pathway [10]. At present, studies have shown that S1PR2 is closely related to the occurrence and development of many tumors, such as pancreatic cancer. [9, 11]、Multiple myeloma [8], colon cancer [12], esophageal adenocarcinoma [13], cholangiocarcinoma [14]. Interestingly, CBA is always accompanied by S1PR2 in the process of playing a role, which attracts our attention [11, 13, 14].

Bile acid (BA), a soluble derivative of cholesterol produced in the liver, is involved in immune responses [15], carcinogenesis, gastrointestinal mucosal barrier function [16–18], and metabolic diseases [19]. Additionally, BA receptor activation can induce the activation of signalling pathways to regulate various physiological functions, i.e., lipid, glucose, and energy metabolism and carcinogenesis [20].Conjugates of chenodeoxycholate becomes the main hydrophobic dihydroxycholate, especially glycodeoxycholate. GCDA has been found to cause cholestasis-related liver injury because of its direct cytotoxicity to hepatocytes [21].Many patients with NAFLD are accompanied by elevated BA levels, which were also observed in an in vivo experiment in a high-fat diet (HF)-NAFLD-HCC animal model [22]. However, the association of NAFLD-HCC with S1PR2 is still unclear.

This study explored the correlation between SIPR2 and the survival of patients by searching online databases. In addition, we investigated the S1PR2 impacts on the biological behaviour of HCC cells and tumour formation in animal models. Mechanistic research was carried out to elucidate the link between GCDA and S1PR2 on the onset and progression of NAFLD-HCC. Finally, the impacts of S1PR2 on the biological behaviour of HCC cells were verified in reverse using JTE-013, an antagonist of S1PR2. Overall, the findings of this study provide novel therapeutic targets for the treatment and prevention of NAFLD-HCC.

## Materials and methods

### Cell culture and reagents

The culturing of HepG2 and Huh7 cells was conducted at a temperature of 37 °C with 5% CO_2_ using DMEM medium containing 1% double antibodies (penicillin–streptomycin mixture) and 10% fetal bovine serum. The cells were observed as anchorage-dependent cells under a microscope. The siRNA sequences of S1PR2 in HepG2 and Huh7 cell lines were as follows: siRNA-1: CCAACAAGGUCCAGGAACA; siRNA-2: GCGCCAUUGUGGUGGAAAA.

The following reagents were used in this study: S1PR2 Antibody (Proteintech, Item No. 21180–1-AP, Wuhan, China), Beta-Actin antibody (CST, Item No. 3700S, USA), SGK1 (D27C11) Rabbit Monoclonal Antibody (CST, Item No. 12103S, USA), PI3 Kinase p85α (6G10) Mouse mAb (CST, Item No. 13666S, USA), PKCα (D7E6E) rabbit monoclonal antibody (CST, Item No. 59754S, USA), Akt Antibody (CST, Item No. 9272S, USA), VEGF-A Antibody (CST, Item No. 65373S, USA), Phospho-Akt (Ser473) (D9E) XP® Rabbit Monoclonal Antibody (CST, Item No. 4060 T, USA), Phospho-mTOR (Ser2448) (D9C2) XP Rabbit Monoclonal Antibody (CST, Item No. 5536 T, USA), JTE-013 (Med Chem Express, Item No. HY-100675, USA) and CBA: Glycochenodeoxycholic Acid (Med Chem Express, Item No. 48305, USA) (concentration: 100umol/L).

### Western blot analysis

Cells were washed 2–3 times with PBS, added with appropriate volume of RIPA lysate (protease inhibitor added minutes before use), scraped off with a cell scratcher, transferred to a 1.5 ml centrifuge tube for a period of 30 min to conduct on-ice lysis, and centrifuged for a period of 10 min at 12000 rpm for the collection the supernatant (total protein solution) at a temperature 4 °C. The measurement of the protein concentration was performed using the BCA Protein Concentration Assay Kit as per the instructions. Afterwards, the protein solution was added to the reduced protein loading buffer, denatured in a boiling water bath for 15 min, and stored in a -20 °C refrigerator. Subsequently, the separation of equal amounts of proteins was carried out using 10% μ-PAGE. The separated proteins were then transferred to PVDF membranes and acted at 200 mA for 2 h. Subsequently, spots in the blots were cut off and bound to antibodies. The membrane blocking was carried out using 5% non-fat milk in TBST for a period of 1 h at room temperature and overnight incubation was conducted at a temperature of 4 °C with primary antibodies.

### Extraction of RNA, reverse transcription and quantitative polymerase chain reaction (RT-qPCR)

The extraction of the cellular RNA was carried out by employing the TRIzol reagent, and cDNA was synthesized by employing the PrimeScrip™ RT kit. Furthermore, RT-qPCR was carried out in triplicate by utilizing the SYBR PreMix Ex Taq™ kit and the ABI 7900HT Real-Time Quantitative Polymerase Chain Reaction System. Following primer sequences were utilized in this reaction: S1PR2: Primer F: 5' TGTATGGCAGCGACAAGAGC 3', Primer R: 5' AGGCAGGACAGTGGAGCAG 3'; GAPDH: Primer F: 5' TCCCATCACCATCTTCCAGG 3', Primer R: 5' GATGACCCCCTTTTGGCTCCC 3'.

### Clone formation

The inoculation of 1 × 10^3^ cells was conducted in six-well plates and incubation was performed at a temperature of 37 °C for 3 weeks, with the solutions changed every three days. Subsequent staining of the plates was performed using 0.2% crystal violet solution at room temperature for a period of 30 min and rinsed three times using PBS. In addition, clones with > 10 cells were counted with a low-magnification microscope. The calculation of the clone formation rate was conducted by using the following equation: clone formation rate = (number of clones/number of inoculated cells) × 100%.

### HCC cell proliferation detected by CCK-8 assay

Various groups of HCC cells were counted under the microscope and a cell suspension was produced at a density of 7 × 10^4^ cells/ml. Furthermore, 100 µl of cell suspension was put into 96-well culture plates. Afterward, three identical wells of each cell type were inoculated in each plate as replicate wells (7 × 10^3^ cells/well, with 100 µl of culture medium as blank control) and incubated overnight at 37 °C. Thereafter, 200 μL of cell suspension was added to 96-well plates and incubation was performed at a temperature of 37 °C for 0, 24, and 48 h. The mixture of Cell Counting Kit -8 (CCK-8) and serum-free Dulbecco's minimum essential medium (BMEM) was produced at a 1:10 volume ratio. Subsequently, the tested sample (100 µL) was added to all the wells and incubation was performed at a temperature of 37 °C for a period of 1 h with 5% CO_2_. Finally, the measurement of absorbance was carried out at a wavelength of 450 nm using a microplate reader.

### Cell wound scratch and transwell assays

The inoculation of cells was performed in a 6-well plate and incubation was conducted for a period of 24 h at 37 °C. Moreover, a sterile tip of a 200 μl micropipette was used to scratch a straight line through the fused monolayer when the cell fusion rate reached 80–90%. Subsequently, we replaced the entire medium with a serum-free medium after washing the floating cells with PBS thrice. A microscope was employed to capture images of the same wound at 0, 24, and 48 h, and ImageJ software was utilized to observe the migration results.

Cell invasion assay was performed at 37 °C using transwell chambers coated with matrigel. The cell density was adjusted at 1 × 10^5^ cells/mL using an FBS-free medium. A total of 200 μL of cells were added to the transwell upper chamber, whereas 800 μL cells containing 10% FBS medium were added to the lower chamber. After incubating for a period of 24 h at 37 °C, methanol was utilized to perform fixation of the cells on the lower surface of the membrane for 20 min and staining was carried out using crystal violet for a period of 30 min. Finally, the number of invading cells in five randomly selected view fields was observed using an inverted light microscope and their images were captured.

### Subcutaneous tumourigenesis assay in nude mice

The approval for the animal experimental protocol involving 4-week-old balb/c nude mice (Hangzhou Ziyuan Experimental Animal Technology Co., Ltd) was acquired from the ethical review committee of Zhongshan Hospital Qingpu Branch, Fudan University, which was in accordance with the Guide for the Care and Use of Laboratory Animals (approval No. 2020–41). Cells were completely digested by trypsin 2 h before transplantation of the tumours, and cell suspensions were prepared by adding appropriate amounts of complete medium containing 10% FBS (cell density: approximately 2 × 10^6^ cells/100 μl). Each mouse was injected subcutaneously with 100ul of previously prepared cell suspensions in the abdominal wall. Six randomly selected experimental mice in each group were killed at the end of feeding by the cervical dislocation method and the tumours were immediately separated by the blunt dissection method. The integrity of the tumour film was maintained during the operation. In addition, the intact tumours in each group were arranged neatly in a certain grouping order using white paper as the background, and a steel ruler was placed under it. Moreover, the weight and volume of the tumours were measured, and images were captured. Subsequently, proteins from some fresh tumour tissues were extracted for RT-qPCR and western blot assays. Finally, some tumours were fixed in formalin for subsequent sectioning and immunohistochemistry.

### Immunohistochemistry assay

Paraffin-embedded sections of xenograft tumours from nude mice (5 μm) were dewaxed in xylene and rehydrated with distilled water in an ethanol gradient. These sections were then incubated with 3% H_2_O_2_ at room temperature for a period of 15 min, blocked with 1% bovine serum albumin, followed by overnight incubation at a temperature of 4 °C with anti-LAGE3 (1:100 dilution; Novus Biologicals, Littleton, CO, USA) or anti-ki67 (ABclonal, Wuhan, China). The subsequent incubation was conducted with horseradish peroxidase-conjugated secondary antibody (1:100 dilution; Thermo Fisher, USA) for a period of one hour at 37 °C. Finally, sections were re-stained with hematoxylin for 3 min after development with 100 μL of 3,3′-diaminobenzidine reagent.

### Bioinformatics related database

The RNAseq data (level3) and corresponding clinical information of liver cancer were obtained from the cancer genome map (TCGA) data set (https://portal.gdc.com)). The differential expression of mRNA was studied by using the Limma package of R software (version 3.40.2). The adjusted P value was analyzed in TCGA or GTEx to correct the false positive results. “Adjusted P < 0.05and log2 (multiple change) > 1 or log2 (multiple change) < −” is defined as the screening of threshold mRNA differential expression. Kaplan–Meier Plotter (http://kmplot.com/analysis/) is mainly used for patient survival analysis and visualization through R-packet ggplot2 [23]. The corrected p < 0.05 was determined to be statistically significant. NAFLD selection criteria: patients with hepatitis virus and alcohol consumption were excluded from this study. According to the results of database sequencing, the patients were divided into high and low expression groups by median method.

### Functional enrichment

To further confirm the underlying function of potential targets, the data were analyzed by functional enrichment. Gene Ontology (GO) is a widely-used tool for annotating genes with functions, especially molecular function (MF), biological pathways (BP), and cellular components (CC). Kyoto Encyclopedia of Genes and Genomes (KEGG) Enrichment Analysis is a practical resource for studying gene functions and associated high-level genome functional information. o better understand the carcinogenesis of mRNA, ClusterProfiler package (version: 3.18.0) in R was employed to analyze the GO function of potential targets and enrich the KEGG pathway. The R software ggplot2 package was used to draw boxplot; the R software pheatmap package was used to draw heatmap.

### Statistical processing

Statistical analysis software SPSS was used to analyze all the experimental data. Use GraphPadPrism to determine the statistical results. All data are expressed as mean ± standard deviation (mean ± SD). T-test was used for statistical analysis of the two groups of data, and one-way ANOVA was used for comparison between groups. *P* < 0.05 is considered to be significant.

## Results

### Association of S1PR2 expression with poor prognosis of patients suffering from NAFLD-HCC

**Fig. 1:**
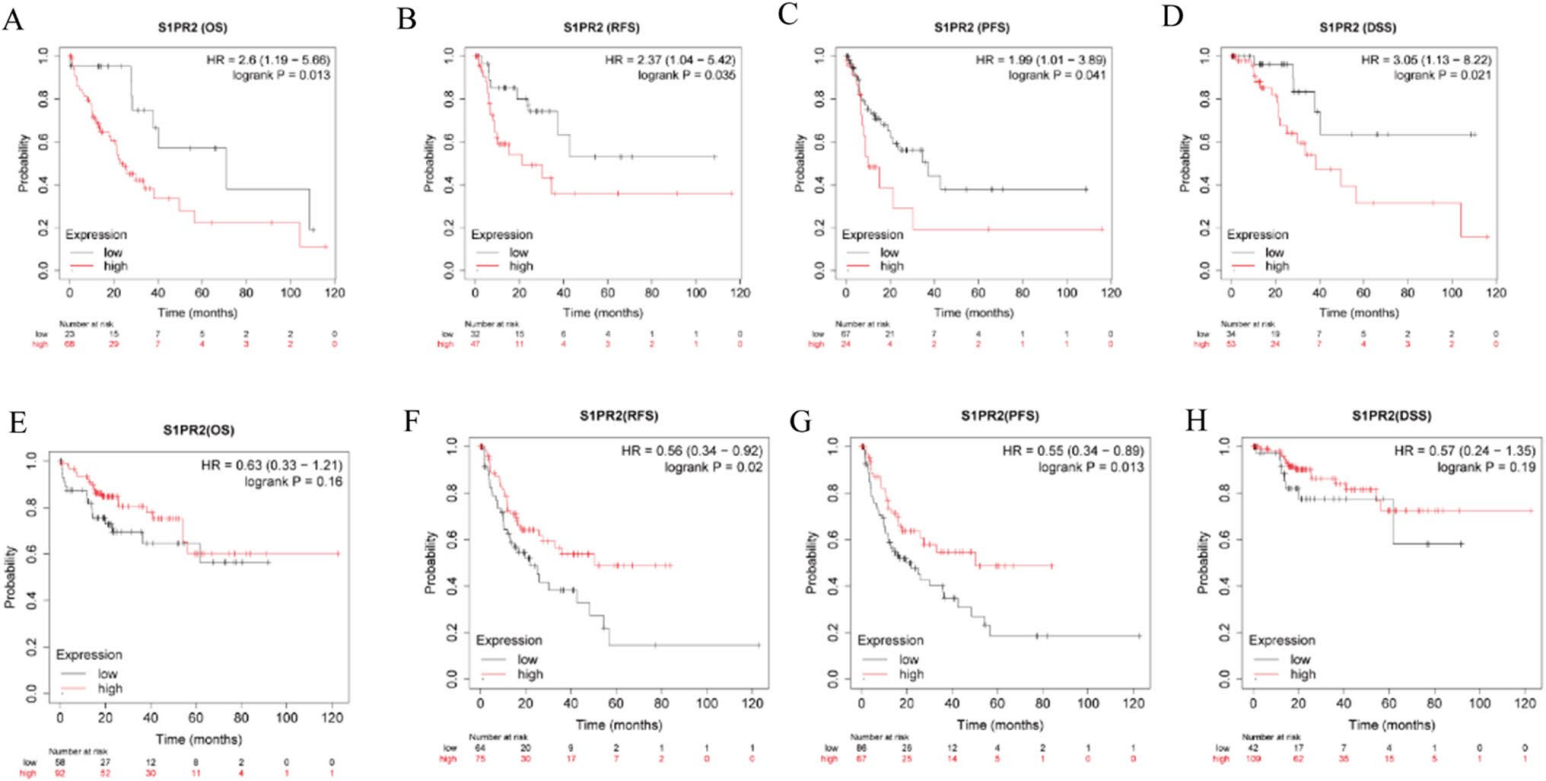
S1PR2 was linked to poor prognosis of NAFLD-HCC patients. **A–D** correlation between S1PR2 and the prognosis of NAFLD-HCC patients in terms of OS, RFS, PFS, and DSS. **E–H** correlation between S1PR2 and the prognosis of hepatitis virus-related HCC patients in terms of OS, RFS, PFS, and DSS

By using the HCC database in Kaplan–Meier Plotter, we investigated the relationship between patient survival and SIPR2 expression. The high-S1PR2 expression group had worse OS, RFS, PFS, and DSS as compared to the low-S1PR2 expression group (Fig. 1A–D). Subsequently, patients infected with the hepatitis virus were reselected for the evaluation of the prognostic relationship between the expression of S1PR2 and the survival of individuals suffering from virus-related HCC. Due to the limitation of the database, the main types of hepatitis viruses in the database are HBV and HCV. S1PR2 expression did not correlate with patient prognosis in terms of OS and DSS, while high S1PR2 expression was a protective factor in the correlation study in terms of RFS and PFS (Fig. 1E–H). Therefore, expression of S1PR2 was linked to poor prognosis of NAFLD-HCC patients.

### S1PR2 promotes the HCC cell development and inhibits their apoptosis

**Fig. 2:**
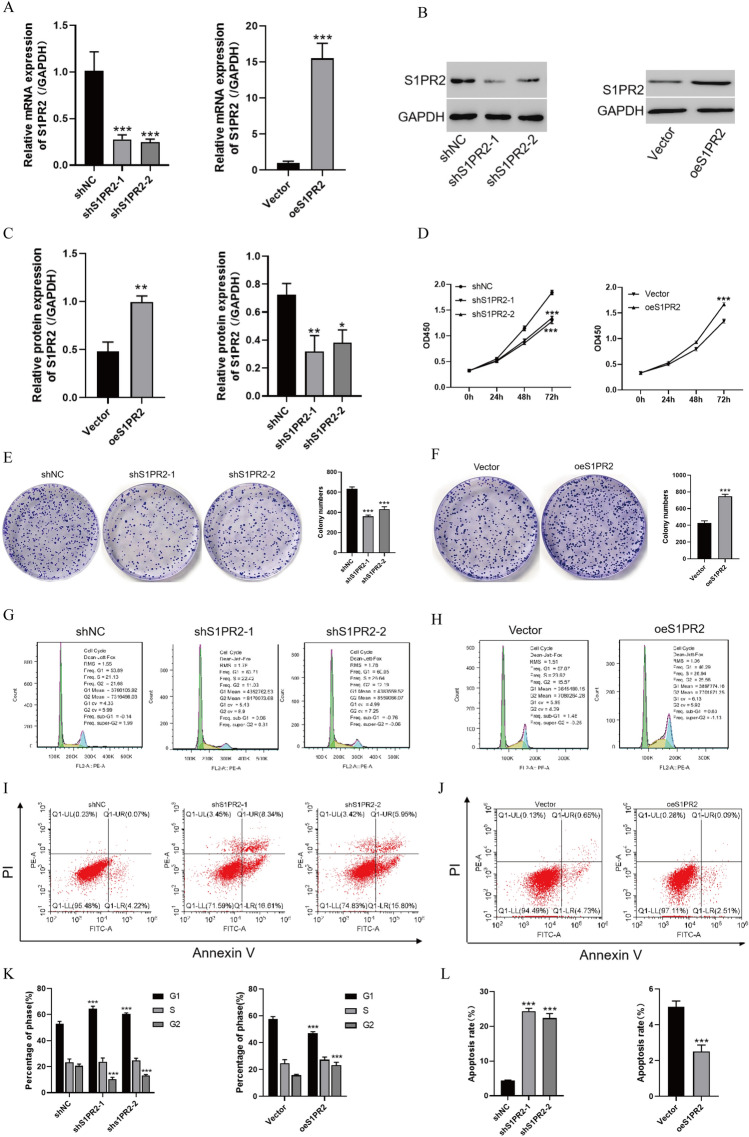
**A** mRNA level of S1PR2 detected by RT-qPCR after S1PR2 plasmid transfection of HCC cells; **B** protein level of S1PR2 detected by western blot after S1PR2 plasmid transfection of HCC cells; **C** statistical difference analysis related to the protein level of S1PR2 revealed by western blot; **D** activity of cells revealed by CCK8; **E**, **F** monoclonal assay for proliferation activity of cells; G and H: flow cytometry for cell cycle change; **I**, **J** flow cytometry for apoptosis; **K** statistical analysis of cell cycle change revealed by flow cytometry; **L** statistical analysis of apoptosis shown by flow cytometry *p < 0.05, **p < 0.01, ***p < 0.001, Vs shNC

The impacts of S1PR2 on the biological activity of HCC cells were evaluated by conducting an experiment in which the S1PR2 gene was knocked down in Huh7 cells but overexpressed in HepG2 cells. The plasmid transfection efficiency was examined using PCR and WB techniques. Both knockdown and overexpression treatments were successful (Fig. 2A–C). Subsequently, the S1PR2 impacts on cell proliferation were evaluated by employing the CCK8 assay (Fig. 2D). Attenuated S1PR2 expression significantly attenuated the proliferative capacity of Huh7 cells, whereas S1PR2 overexpression significantly promoted the proliferative capacity of HepG2 cells. However, suppressed S1PR2 expression significantly attenuated the clonogenic ability of Huh7 cells, whereas S1PR2 upregulation promoted the clonogenic ability of HepG2 cells (Fig. 2E, F).

In addition, cell cycle analysis suggested that S1PR2 downregulation increased the cell percentage in the G1 phase and reduced it in the G2 phase. In contrast, S1PR2 overexpression reduced the cell percentage in the G1 phase and improved it in the G2 phase (Fig. 2G, H, and K). These results suggest that S1PR2 promoted the HCC cell development by enhancing the G1/G2 phase transition.

The effect of S1PR2 on the apoptotic rate of HCC cells was further evaluated using flow cytometry and staining assays. Reducing S1PR2 expression increased the early and late apoptotic index of Huh7 cells. In contrast, high S1PR2 expression significantly decreased the early apoptosis rate of HepG2 cells (Fig. 2I, J and L). Therefore, S1PR2 can inhibit apoptosis in HCC cells.

### S1PR2 promotes migration and invasion of HCC cells

**Fig. 3:**
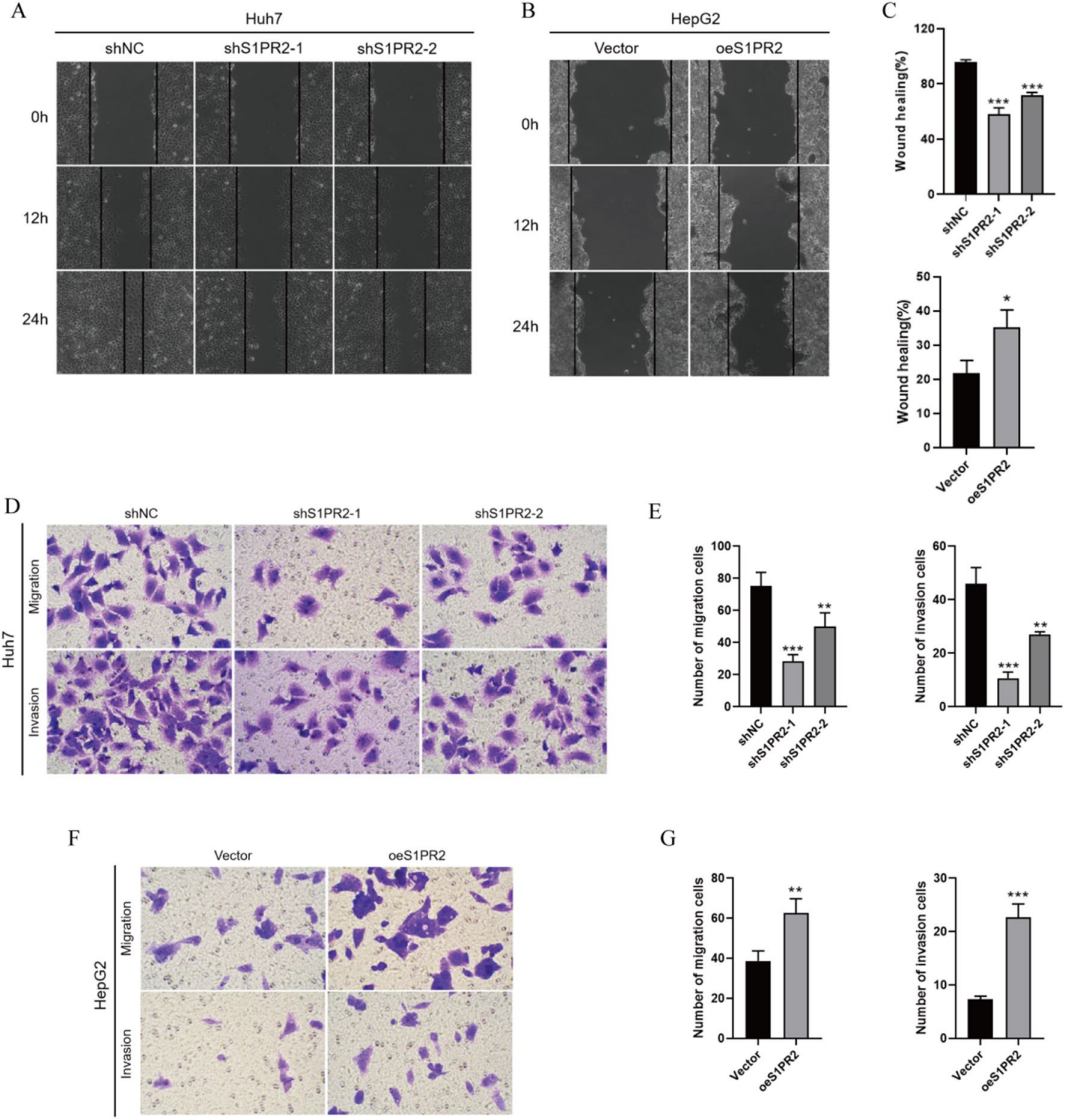
**A**, **B**, and **C** Migration effect of S1PR2 on HCC cells and statistical analysis of related migration rate. **D**, **E** Migration and invasion of cells detected by Transwell after transfection of Huh7 cells with S1PR2 interference plasmid and related statistical analysis; **F**, **G** Migration and invasion of cells detected by Transwell after transfection of HepG2 cells with S1PR2 overexpression plasmid and related statistical analysis; **p < 0.01, ***p < 0.001

For the evaluation of cell migration and invasion ability, scratch and transwell assays were conducted, thus elucidating the role of S1PR2 in the malignant phenotype of HCC cells. S1PR2 downregulation attenuated the migration of Huh7 cells, whereas S1PR2 overexpression displayed contrasting results (Fig. 3A–C). According to the transwell assay, S1PR2 knockdown significantly reduced the invasive ability of Huh7 cells, whereas the invasive ability of HepG2 cells was enhanced by S1PR2 overexpression (Fig. 3D–G). These results suggest that S1PR2 promoted the invasion and migration of HCC cells.

### S1PR2 promotes in vivo growth of HCC xenograft tumours

**Fig. 4:**
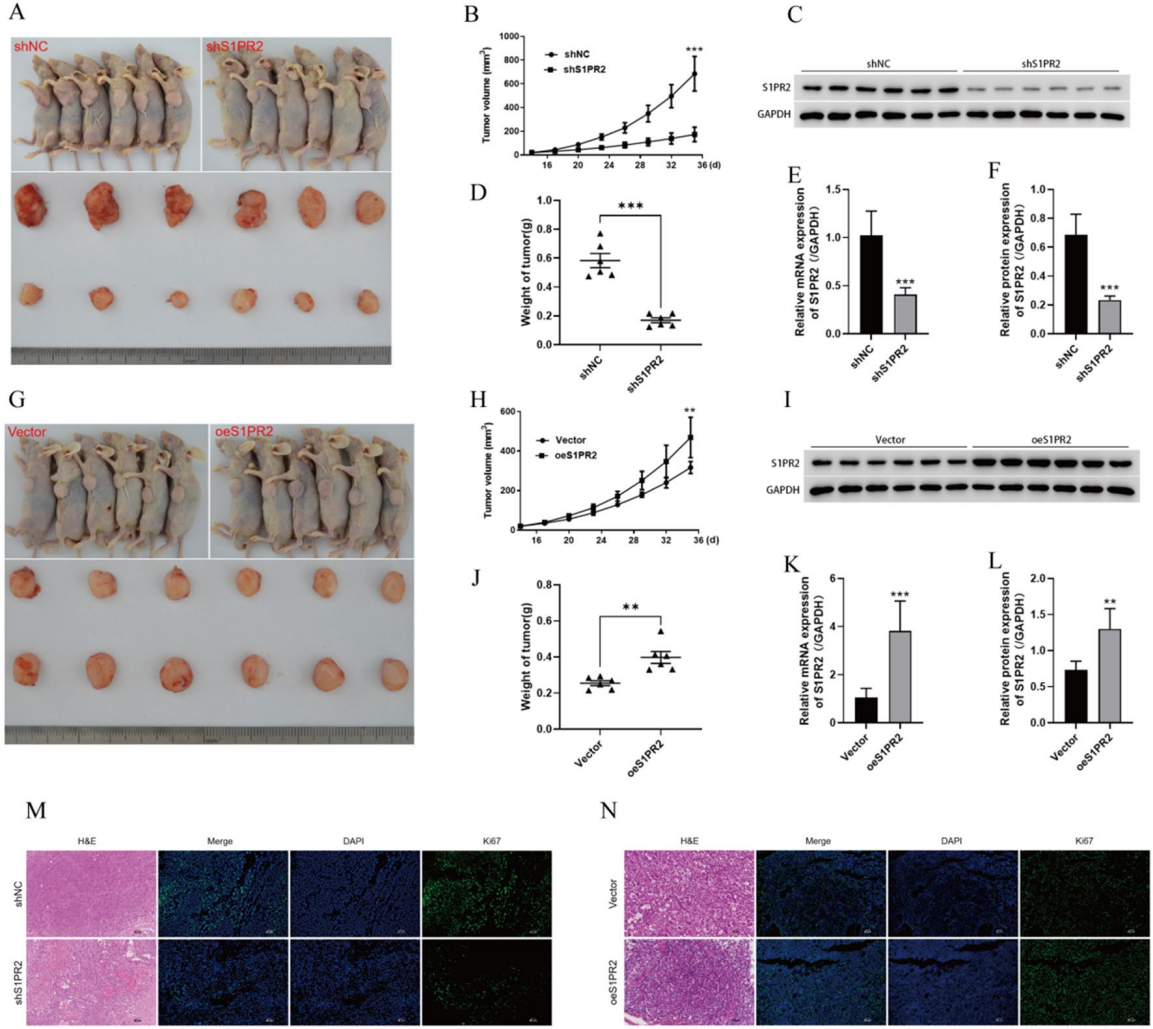
Interfering with S1PR2 inhibits the growth of HCC tumours. **A** sh-NC and sh-S1PR2 expression in the in vivo and ex vivo tumours in nude mice; **B** subcutaneous tumour growth curves in sh-NC and sh-S1PR2 groups; **C** S1PR2 protein levels in tumour tissues observed via western blot; **D** weight of tumours; **E** S1PR2 levels of mRNA in tumour tissues observed by employing RT-qPCR; **F** statistical analysis of protein levels of S1PR2 in relevant tissues, where S1PR2 can promote the development of HCC tumour cells; **G** In vivo and ex vivo tumours in nude mice in Vector and oeS1PR2 groups; **H** subcutaneous tumour growth curves of Vector and oeS1PR2 groups; **I** levels of protein of S1PR2 in tumour tissues observed using western blot; **J** weight of tumours; **K** Levels of mRNA of S1PR2 in tumour tissues revealed by RT-qPCR; **L** statistical analysis of protein levels of S1PR2 in relevant tissues; **M**, **N** pathological changes of tumour tissues observed by H&E staining; proliferation of tumour cells detected by Ki67 immunofluorescence staining. **p < 0.01, ***p < 0.001

As per the aforementioned results, S1PR2 can perform an essential function in the in vivo growth of HCC tumours. A nude mouse tumourigenic model was constructed using Huh7 and HepG2 (Fig. 4A and G). Cells transfected with sh-S1PR2 produced significantly smaller tumours as compared to cells transfected using sh-NC. Quantification of tumour weight and volume indicated that tumours in the sh-NC group were considerably larger in comparison to the ones in the sh-S1PR2 group (Fig. 4B and D). Additionally, the levels of mRNA and protein of S1PR2 were substantially lower in the tumours of mice in the sh-S1PR2 group (Fig. 4C, E, and F). The growth curves suggested that the rate of tumour growth was notably slower in the sh-S1PR2 group (Fig. 4B). According to the comparative results of tumourigenic growth between the Vector and oeS1PR2 groups, the subcutaneous tumour volume, mass, and S1PR2 expression of tumours were substantially higher in the oeS1PR2 group as compared to the Vector group (Fig. 4H–L). Silencing of S1PR2 decreased ki67 expression in tumours, whereas increasing S1PR2 expression had a contrasting effect (Fig. 4M, N). In summary, S1PR2 promoted the in vivo growth of HCC tumours.

### S1PR2 antagonist JTE-013 significantly inhibits the proliferation and invasive ability of HCC cells

**Fig. 5:**
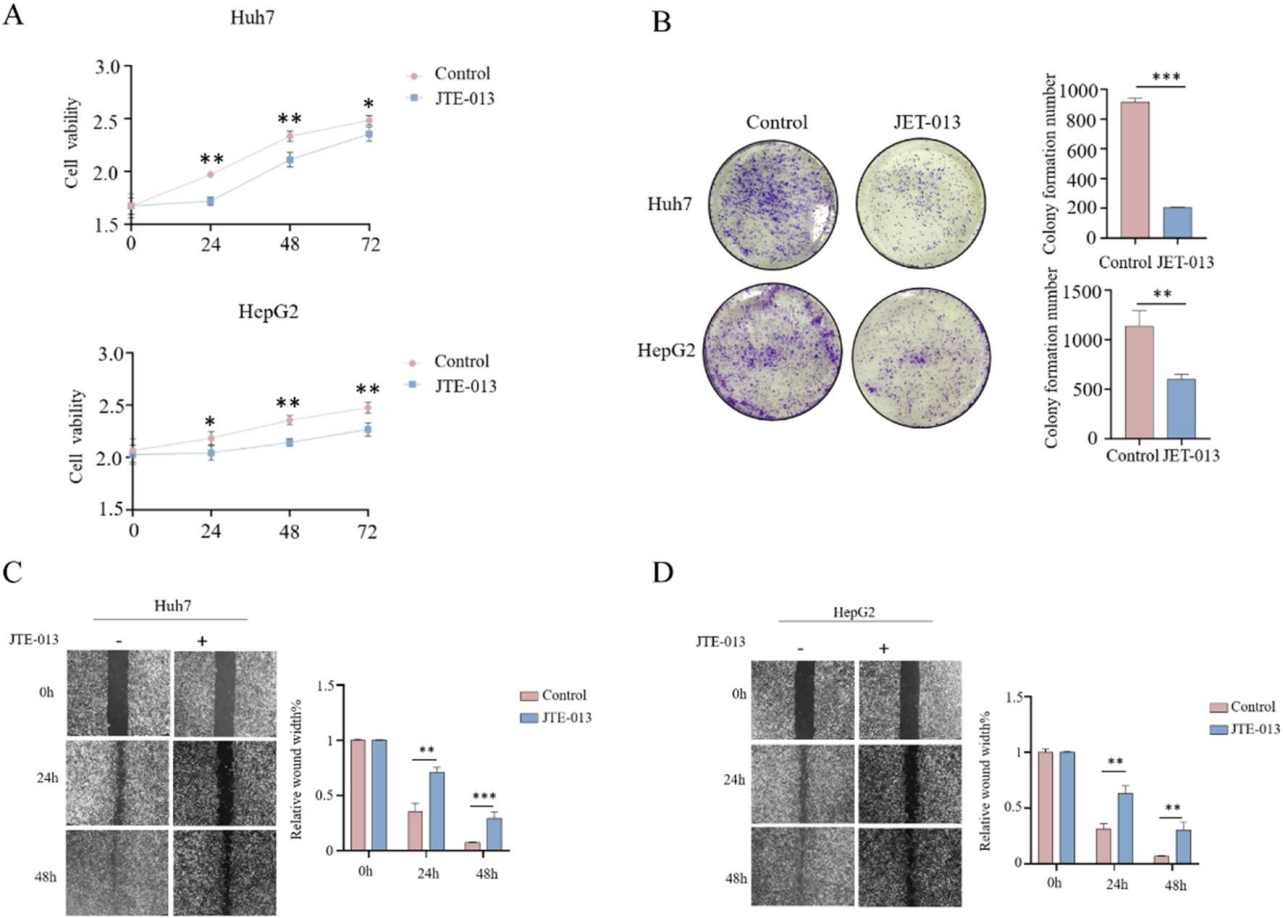
Biological activity assay of HCC cells after antagonism of S1PR2 expression by JTE-013. **A** Cell activity measurement by the CCK8 assay; **B** cell clone formation assay; **C**, **D** cell scratch assay

The treatment of HCC cells with JTE-013, the antagonists of S1PR2, verified the biological effects of S1PR2 on HCC cells (Fig. 5). CCK8 assay findings indicated that JTE-013 could significantly inhibit the growth rate of HCC, Huh7, and HepG2 cells (Fig. 5A). As per the clone formation assay, JTE-013 significantly inhibited the proliferation ability of HCC cells (Fig. 5B). Moreover, the scratch assay confirmed JTE-013’s significant inhibitory effects on the migration ability of HCC cells (Fig. 5C, D). Therefore, antagonizing S1PR2 expression significantly inhibited the malignant phenotype of HCC cells.

### S1PR2 enhances HCC cell proliferation through activation of the PI3K/AKT/mTOR signalling pathway

**Fig. 6:**
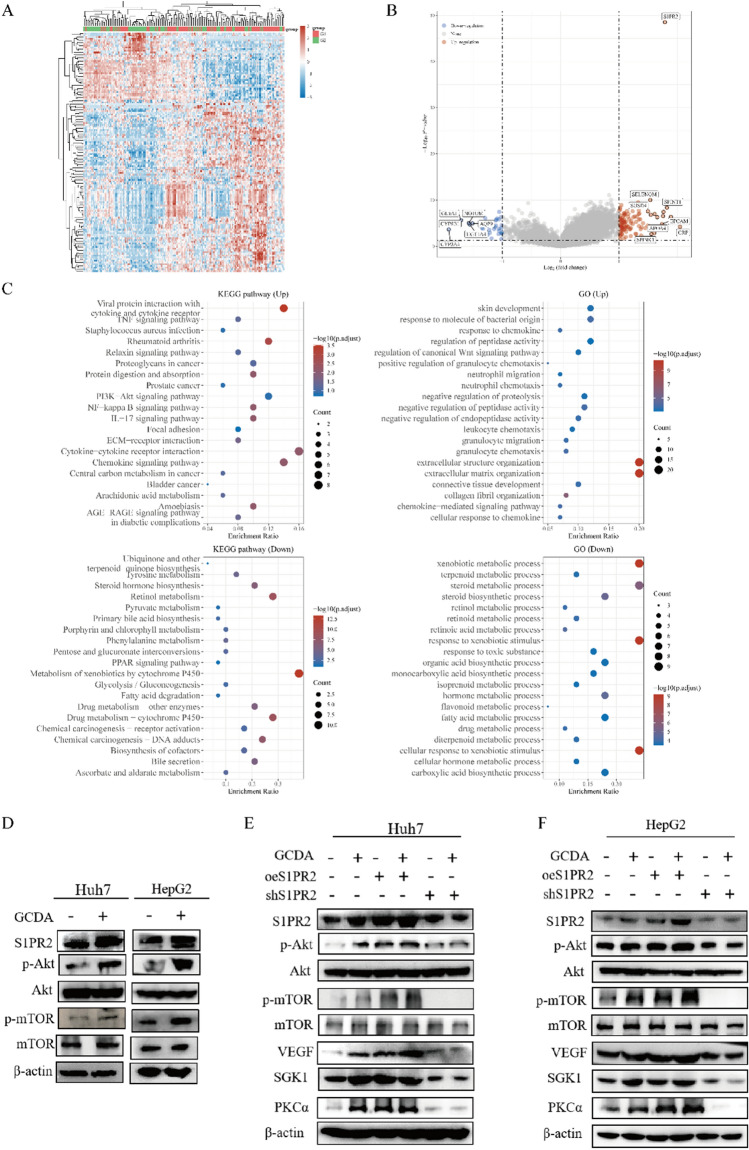
GCDA regulates the PI3K/AKT/mTOR signalling pathway by promoting S1PR2 expression. **A** heat map of differential expression between low- and high- S1PR2 expression groups; **B** volcano plot revealing differential expression between low- and high- S1PR2 expression groups; **C** functional enrichment results of genes with differential expression between low- and high- S1PR2 expression groups. **D**–**F** The levels of protein of the PI3K/AKT/mTOR pathway in HCC cells with knocked-down and overexpressed S1PR2 in the presence or absence of GCDA conditions detected by western blot

RNA-seq datasets of HCC samples from TCGA were collected to investigate the molecular mechanisms of S1PR2 involvement in HCC progression. GO and KEGG functional enrichment analyses were conducted on the relevant datasets after removing data from patients with a history of alcohol consumption and viral hepatitis. The results revealed that the PI3K/AKT/mTOR signalling pathway was significantly enriched in the high-S1PR2 expression group (Fig. 6A–C). In addition, the correlation between CBA, S1PR2, and the PI3K/AKT/mTOR pathway was explored. Subsequently, western blot analysis was performed for Huh7 and HepG2 cells. CBA significantly promoted S1PR2, p-AKT, and p-mTOR expression in both Huh7 and HepG2 cells (Fig. 6D). Furthermore, protein expression levels of p-AKT, p-mTOR, VEGF, SGK1, and PKCα were substantially increased in the S1PR2 overexpression group in comparison to the S1PR2 knockdown group (Fig. 6E, F). GCDA administration further enhanced the expression levels of p-AKT, p-mTOR, VEGF, SGK1, and PKCα. Moreover, both the presence and absence of CBA did not reveal any significant change in the levels of S1PR2, p-AKT, p-mTOR, VEGF, SGK1, and PKCα proteins under S1PR2 knockdown, indicating that GCDA regulates the PI3K/AKT/mTOR pathway by mediating S1PR2 expression (Fig. 6E, F). These results suggest that GCDA could enhance S1PR2 expression and activate the PI3K/AKT/mTOR signalling pathway, thus promoting the growth of HCC cells.

## Discussion

HCC poses a great threat to human health due to its high malignancy and aggressive nature. It can be classified into various types according to the aetiology, and the onset mechanism differs across different types. NAFLD is one of the major causes of HCC, and it is one of the most prevalent chronic liver diseases with an overall prevalence rate of 25% around the globe [24, 25]. Furthermore, the NAFLD prevalence has exceeded 30% in the United States, and the increase in obesity and metabolic syndrome will result in a further increase [25]. Individuals suffering from NAFLD have a high probability of developing HCC [26], which is linked to the poorer OS in these patients [27]. CBA can promote the aggressive development of esophageal adenocarcinoma cells and cancer stem cell proliferation by regulating S1PR2 levels [13]. The association of CBA with the onset of NAFLD and HCC has been confirmed by several studies [22, 28]. Therefore, this research investigated the correlation between GCDA, S1PR2, and NAFLD-HCC to present novel understandings of the prevention and treatment of NAFLD-HCC.

In this study, the association of high S1PR2 expression with poor prognosis in individuals suffering from NAFLD-HCC was confirmed using bioinformatics analysis. However, S1PR2 expression was not associated with patient prognosis and even served as a protective factor in viral hepatitis-related HCC. It is conjectured that S1PR2 performs an essential function in the onset and advancement of NAFLD-HCC, as demonstrated by cellular and animal experiments. A high expression level of S1PR2 promotes the viability, proliferation, metastasis, and invasion of HCC cell lines. Additionally, in vivo tumourigenesis experiments in mice have demonstrated the role of S1PR2 as an oncogene in the onset and progression of NAFLD-HCC. Furthermore, the role of S1PR2 in tumor is still controversial. Some studies have found that S1PR2 knockout can accelerate the progression of NASH to HCC [29], but our research shows that it plays the role of oncogenes. In addition, studies have shown that S1PR2 served as a cancer-promoting gene in papillary thyroid carcinoma cells [30], multiple myeloma [8], chronic granulocytic leukemia [31], bladder cancer, and prostate cancer [32], but served as a cancer-suppressing gene in melanoma [33], glioblastoma [34], and diffuse large B-cell lymphoma [35].

Moreover, the antagonist of S1PR2, JTE-013 [6], was used to verify our conjecture again. JTE- 013 can express S1PR2 while decreasing the degree of malignant phenotypes such as cell viability, proliferation, and invasive metastasis of HCC cells. Thus, S1PR2 is associated with the onset and progression of HCC, and blocking its expression reduces the malignant biological phenotype of HCC cells.

The mechanism via which S1PR2 served as an oncogene was further explored and bioinformatics enrichment analysis highlighted the relevance of S1PR2 to multiple mechanisms. As we can see, S1PR2 is closely related to metabolism. According to GO/KEGG analysis, we can find that central carbon metabolism in cancer, arachidonic acid metabolism, retinol metabolism, primary bile acid biosynthesis, metabolism of xenobiotics by cytochrome P450, xenobiotic metabolic process and other pathways are related, which indicates that the changes of metabolic pathway may play an important role in the occurrence and development of NAFLD-HCC, and S1PR2 may play a certain role in this process, which is consistent with previous studies [36–39]. Overexpression of S1PR2 activated the PI3K/AKT/mTOR pathway, which can be associated with the malignant phenotype of HCC cells. However, silencing S1PR2 significantly inhibited the PI3K/AKT/mTOR pathway, which regulates the biological activity of HCC cells. Additionally, GCDA regulates the activity of the PI3K/AKT/mTOR pathway by increasing the expression level of S1PR2, thus promoting the onset and progression of NAFLD-HCC and enhancing the malignant biological activity of HCC cells. Moreover, the PI3K/AKT/mTOR is a classical signalling pathway involved in various cellular activities such as involvement in angiogenesis, thereby promoting cell proliferation, invasion, migration, apoptosis, and metabolism [40–43]. In summary, the PI3K/AKT/mTOR pathway performs a significant function in tumour formation and is closely associated with the onset and advancement of malignant tumours [44].

CBA is involved in the onset and progression of NAFLD-HCC [28]. This pathogenesis is different from that of hepatitis B-related HCC. CBA can activate S1PR2 [14, 45, 46], increase the level of S1P in the nucleus, upregulate genes encoding nuclear receptors, lipid metabolism, and glucose metabolism-related enzymes [47], and ultimately regulate multiple biological activities. According to the results of this study, GCDA promotes S1PR2 expression, activates the PI3K/AKT/mTOR pathway, and enhances the proliferation, invasion, and migration of HCC cells.

In conclusion, S1PR2 is correlated with the onset and advancement of NAFLD-HCC, and its high expression is the main reason for a poor prognosis. Cellular and in vivo assays demonstrated the association of S1PR2 expression with the malignant phenotype of HCC cells. GCDA activates the AKT/mTOR pathway by promoting S1PR2 expression and accelerates NAFLD-HCC progression. However, there are some limitations in this study. The inclusion of eligible NAFLD-HCC patients from the database by only removing patients with a history of virulent hepatitis and excessive alcohol consumption resulted in some bias. Despite the need for further improvement of the experiment, the results of this research present novel insights for the prevention and treatment of NAFLD-HCC.

## Data Availability

The data underlying this article will be shared on reasonable request to the corresponding author.
